# HOTAIRM1 Promotes Malignant Progression of Transformed Fibroblasts in Glioma Stem-Like Cells Remodeled Microenvironment *via* Regulating miR-133b-3p/TGFβ Axis

**DOI:** 10.3389/fonc.2021.603128

**Published:** 2021-03-19

**Authors:** Haiyang Wang, Haoran Li, Qianqian Jiang, Xuchen Dong, Suwen Li, Shan Cheng, Jia Shi, Liang Liu, Zhiyuan Qian, Jun Dong

**Affiliations:** Department of Neurosurgery, The Second Affiliated Hospital of Soochow University, Suzhou, China

**Keywords:** glioma stem-like cells, fibroblasts, tumor microenvironment, HOTAIRM1, malignant transformation

## Abstract

Recent studies have reported that cancer associated fibroblasts (CAFs) and glioma stem-like cells (GSCs) played active roles in glioma progression in tumor microenvironment (TME). Long non-coding RNAs (lncRNAs) have been found to be closely associated with glioma development in recent years, however, their molecular regulatory mechanisms on CAFs in GSCs remodeled TME kept largely unelucidated. Our study found that GSCs could induce malignant transformation of fibroblasts (t-FBs) based on dual-color fluorescence tracing orthotopic model. Associated with poor prognosis, Lnc HOXA transcript antisense RNA, myeloid-specific 1 (HOTAIRM1) was highly expressed in high-grade gliomas and t-FBs. Depleting HOTAIRM1 inhibited the proliferation, invasion, migration, and even tumorigenicity of t-FB. Conversely, overexpression of HOTAIRM1 promoted malignancy phenotype of t-FB. Mechanistically, HOTAIRM1 directly bound with miR-133b-3p, and negatively regulated the latter. MiR-133b-3p partly decreased the promotion effect of HOTAIRM1 on t-FBs. Furthermore, transforming growth factor-β (TGFβ) was verified to be a direct target of miR-133b-3p. HOTAIRM1 can modulate TGFβ *via* competing with miR-133b-3p. Collectively, HOTAIRM1/miR-133b-3p/TGFβ axis was involved in modulating t-FBs malignancy in TME remodeled by GSCs, which had the potential to serve as a target against gliomas.

## Introduction

Gliomas are the most prevalent primary intrinsic tumors in the central nervous system, and glioblastoma multiforme (GBM) accounts for nearly half of gliomas (1–3). Although the treatment is constantly optimized (4), GBM is regarded as one of the most malignant and aggressive cancer, which is characterized of poor prognosis and low overall survival. Recently, some studies have focused on the interactions between GSCs and stromal cells in TME, which is regarded as crucial element for glioma progression recently (5).

Among the stromal cells in TME, fibroblasts are key components involved in progression and metastasis of various malignancies (6, 7). Chronic activation of peri-tumor fibroblasts by cancer cells leads to formation of a subgroup of cells generally known as cancer associated fibroblasts (CAFs) (8). As a significant crosstalk mediator between cancer and stroma in TME, fully exploring the roles of CAFs may help to develop new possible therapeutic approaches against gliomas (9). Local accumulation of CAFs in TME is related with poor prognosis (10, 11), CAFs can promote the proliferation and metastasis of breast cancer (12), endometrial cancer (13), adenocarcinoma of esophagogastric junction (14), melanoma (15), gastric cancer (16), etc. However, the roles of fibroblasts in gliomas TME, and their interactions or crosstalk with GSCs have not been fully elucidated up to now.

LncRNAs are a group of non-coding RNAs with over 200 nucleotides in length (17), which could activate/silence target gene on the level of transcription and/or post-transcription level (18). Emerging studies have declared that aberrant expression of lncRNAs is ubiquitous and relevant with multiple malignant phenotypes of tumors (19–21). LncRNA HOTAIRM1 is located in the HOX gene cluster and regulates the family of HOXA genes (22), which is associated with tumor progression, including colorectal (23), gastric (24), lung cancers (25), and pancreatic cancer (26), et al. However, the role of HOTAIRM1 played in interactions between GSCs and stromal cells in glioma microenvironment has never been reported previously.

Based on the findings that *in vivo* malignant transformation of fibroblasts with HOTAIRM1 upregulation after interaction with GSCs, further exploration the roles of HOTAIRM1 initiated molecular regulatory pathway on proliferation, invasion, migration, and tumorigenicity of t-FBs, will provide new experimental references for potential treatment strategy targeting on t-FBs in glioma microenvironment.

## Material and Methods

### Tumor Specimens, Experimental Animals, and Cell Culture

Surgical specimen from glioma patients were collected from the Department of Neurosurgery, the Second Affiliated Hospital of Soochow University with informed consent. The whole process obeyed the rules of the Ethics Committee of the Second Affiliated Hospital of Soochow University.

Balb/c nude mice expressing green fluorescent protein (GFP) were bred in specific pathogen free (SPF) experimental animal center in Soochow University (27). All of the animal studies adhered to the rules of the Ethics Committee of the Second Affiliated Hospital of Soochow University.

The GSCs-SU3 cells derived from surgical specimen of glioma patient (28) and were cultured in DMEM/F12 medium favored for neural stem cell growth (Gibco, USA) including 1x B27 Supplement (Gibco, USA), 20 ng/ml epidermal growth factor (EGF) (Gibco, USA), and 20 ng/ml basic fibroblast growth factor (bFGF) (Gibco, USA). The normal human astrocyte cells (NHA), glioma cell lines (SNB19, A172, U343) were cultured in DMEM (Hyclone, USA) containing 10% fetal bovine serum (FBS) (BI, Israel). All cells were cultured in the incubator (SANYO, JP) at 37°C with 5% CO_2_.

### Establishment of Dual-Color Fluorescence Tracing Orthotopic GSCs Model

Human GSCs-SU3 cells (28) with red fluorescence protein (RFP) stable expression *via* lentivirus transfection (SU3-RFP) (**Supplementary Figures 1A, B**). GSCs-SU3RFP cells (1 × 10^6^) were injected slowly into the caput nuclei caudate of 4 weeks old GFP Balb/c nude mice (15–20 g) with stereotaxic techniques (**Supplementary Figure 1C**). All mice were sacrificed 4 weeks later under general anesthesia, and the xenografts were harvested, fine minced, and digested with trypsin to prepare single-cell suspension. GSCs derived tumor cells (RFP+) and host derived tumor stromal cells (GFP+) in the xenograft parenchyma can be distinguished under fluorescence microscopic view (**Supplementary Figures 1D–F**). GFP+ cells with high proliferative capacity were mono-cloned with micro-pipetting techniques (**Supplementary Figures 1G, H**), and continued subculturing (**Supplementary Figures 1I, J**). Two of the mono-cloned GFP+ cells (positive for fibroblast makers α-SMA, vimentin, and S100A4) with unlimited proliferation ability (**Supplementary Figures 1K–M**), were named after transformed fibroblasts t-FB1 and t-FB2, respectively.

### CCK8 Assay

Cells were seeded into 96-well plates at a density of 3,000 cells/well in 100 µl DMEM. Ten µl CCK8 reagent (Dojindo, Japan) was added into each well every 24 h and incubated for another 2 h at 37°C. Absorbance value at 450 nm was recorded with a spectrophotometer (Tecan, Switzerland).

### Immunocytochemical Staining

Transformed fibroblasts were fixed with methanol for 20 min, then permeabilized with 0.25% Triton X-100 (Beyotime, China), and incubated in blocking solution for 1 h. The primary antibodies of α-SMA, vimentin, and S100A4 (CST, USA) were applied for 1 h, respectively. After washing with PBS for three times, the second antibody (Beyotime, China) was applied for 30 min. Finally, the cells were stained with diaminobenzidine (DAB) and hematoxylin, observed under fluorescence microscope (Zeiss, Germany) at a magnification of 200×.

### Vector Construction and Cell Transfection

The short hairpin RNA (shRNA) targeting HOTAIRM1 (shHOTAIRM1-1 and shHOTAIRM1-2), the overexpression vector of HOTAIRM1, TGF-β, and the corresponding negative control, the miR-133b-3p mimics, inhibitors, and corresponding negative control, were all designed by GenePharma (Shanghai, China). The corresponding vectors and their controls were transfected into transformed fibroblasts according to the manufacturer’s protocol.

### Quantitative Real-Time Reverse Transcription PCR (qRT-PCR)

Total RNA was extracted with TRIzol (Invitrogen, USA). qRT-PCR was performed to evaluate the expression levels of HOTAIRM1, miR-133b-3p, and TGF-β. The expression of U6 and GAPDH was applied as control. The expression level was analyzed with 2^−ΔΔCt^ method.

### 5-Ethynyl-20-Deoxyuridine (EdU) Assay

Cells (5 × 10^4^) were seeded in 24-well plates and cultured overnight. Then 300 µl EdU (50 µM)(RiboBio, China) was added in each well and incubated for 2 h. Then cells were fixed by 4% paraformaldehyde for 20 min and permeabilized by 0.5% TritonX-100 (Beyotime, China) for 20 min, dyed in 300 µl Apollo dye solution (RiboBio, China) for 25 min. Cell nuclei were dyed with Hoechst (RiboBio, China) for 10–30 min. Cells were observed under an inverted fluorescence microscope (Zeiss, Germany) at a magnification of 200×. And then proportion of EdU positive cells was calculated.

### Invasion Assay

Transwell chambers (Corning, USA) were coated with Matrigel (dilution 1:8; BD, USA). Then 120 μl serum-free medium containing 5 × 10^4^ cells was added in the upper chamber, 600 μl complete medium containing 10% FBS was added in the lower chamber, then cultured for 24 h at 37°C. The cells on the upper surface were erased with cotton swabs. The cells on the lower surface were fixed with methanol for 30 min and stained with 0.1% crystal violet, cell images were captured with an inverted microscope (AMG, USA) at a magnification of 200×.

### Wound Healing Assay

Cells were seeded on six-well plate and cultured at 37°C overnight. A 200 μl pipette tip was applied to make wounds on monolayer cells. Cells were washed by PBS and cultured in DMEM without FBS. The cells were imaged with an inverted microscope (AMG, USA) at a magnification of 100×. Images of the wound area were analyzed *via* Image J software after 24 h (NIH, Bethesda, USA).

### Luciferase Reporter Assay

The HOTAIRM1 fragment with the miRNAs binding sequences were inserted into the pMIR-REPORT vectors. Similarly, the 3′-UTR fragments of TGF-β with the miRNAs binding sequence were also inserted into pMIR-REPORT vectors. Transformed fibroblasts were transfected with the corresponding miRNAs and the reporter vectors. The mutated vectors were used as control. The duration of transfection was 36 h. Luciferase activity was evaluated by the Dual Luciferase Reporter Assay System (Promega, USA).

### Western Blot

Total cell proteins were extracted with RIPA buffer (Beyotime Biotechnology, China). Twenty μg total proteins were separated by 10% SDS-PAGE and transferred to PVDF membrane, then incubated with the primary antibodies against TGF-β (1:1,000, CST, USA) and GAPDH (1:5,000, CST, USA) overnight. Then the membrane was incubated with the secondary antibody for 1 h. ECL method was used for visualization for quantitative analysis.

### Enzyme Linked Immunosorbent Assay (ELISA)

Sample and the diluent buffer were added to the wells of the 96-well plate which was pre-coated with anti-TGF-β antibody (Abnova, USA), then shake the plate gently to mix thoroughly, and incubate at 37°C for 30 min. Discard the solution in the plate and fill each well completely with Wash Buffer (1×) and vortex gently on the shaker for 2 min. Repeat this procedure four more times, 50 μl HRP conjugated anti-TGF-β antibody was added into each well. Incubate at 37°C for 30 min, then wash the plate for three times, following with adding 50 μl TMB chromogenic reagent A and B, vortexing gently the plate on the shaker for 30 s, and incubating in dark at 37°C for 15 min, finally adding 50 μl Stop solution into each well and mix thoroughly. The absorbance at 450 nm was detected with a spectrophotometer (Tecan, Switzerland).

### Tumorigenicity Assay

Athymic Balb/c nude mice (4 weeks old, 15–20 g) were bred in the SPF animal center. Then 1 × 10^6^ transformed fibroblasts with up/downregulation of HOTAIRM1 and the corresponding negative control were injected subcutaneously into the right flank of each mouse, respectively. After 5 weeks, all mice were sacrificed under general anesthesia, and the tumors were excised and weighed.

### Statistical Analysis

All data were expressed as mean ± SD and analyzed with t-test, one-way ANOVA, or two-way ANOVA using the software of Prism 7.0 (GraphPad Software, USA). P-value <0.05 was considered statistically significant (*p < 0.05; **p < 0.01; ***p < 0.001; **** p < 0.0001).

## Results

### Overexpression of LncRNA HOTAIRM1 in Glioma Tissue/Cell Lines and Malignant Transformed Fibroblasts

**Figure 1:**
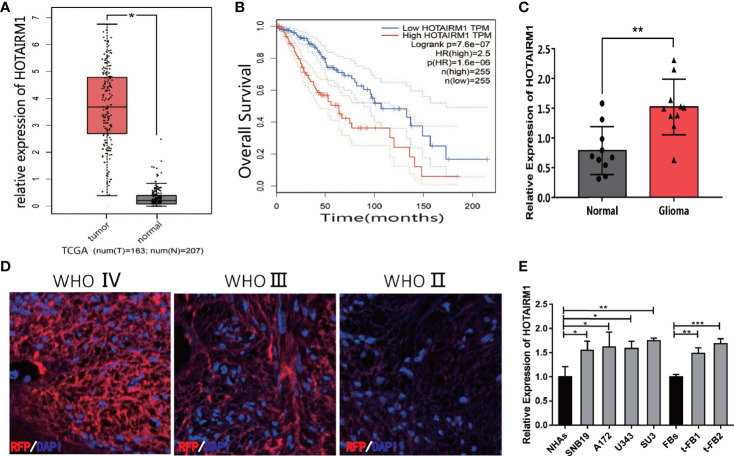
Expression of HOTAIRM1 in malignant transformed fibroblasts (t-FBs) and glioma surgical specimens and cell lines. **(A)** HOTAIRM1 expression level between glioma and normal brain tissue in TCGA. *p < 0.05, Student’s t test. **(B)** Overall survival rate of glioma patients in low HOTAIRM1 group and high HOTAIRM1 group. **(C)** HOTAIRM1 expression level in clinical glioma specimen (n = 10) and paired normal brain tissue (n = 10). **p < 0.01, Student’s t test. **(D)** HOTAIRM1 expression level in clinical specimen with different malignancy grade by RNA FISH assay. **(E)** HOTAIRM1 expression level in malignant transformed fibroblasts (t-FB), normal fibroblasts, glioma cell lines, and GSCs. *p < 0.05, **p < 0.01, ***p < 0.001, one-way ANOVA.

HOTAIRM1 expression in gliomas was evaluated in The Cancer Genome Atlas (TCGA) database, indicating HOTAIRM1 upregulation in gliomas compared with adjacent normal brain tissue (**Figure 1A**). TCGA survival curve analysis of gliomas showed that the survival of patients with high HOTAIRM1 expression decreased significantly (**Figure 1B**). Clinical glioma samples (n = 10) were collected to further verify HOTAIRM1 expression, which was in accordance with the TCGA results (**Figure 1C**). RNA fluorescence *in situ* hybridization (FISH) demonstrated that HOTAIRM1 expression was positive correlated with glioma malignancy grades in tissues sections of clinical specimens (**Figure 1D**). In addition, HOTAIRM1 expression was detected in the malignant transformed fibroblasts (t-FB1 and t-FB2), normal fibroblasts, glioma cell lines (SNB19, A172, U343), GSCs-SU3, and normal human astrocytes (NHAs), which disclosed HOTAIRM1 upregulation in glioma cell lines, GSCs, and transformed fibroblasts, when compared with NHAs and normal fibroblasts (**Figure 1E**).

### HOTAIRM1 Downregulation Inhibited Proliferation, Invasion, Migration, and Tumorigenesis of Malignant Transformed Fibroblasts

**Figure 2:**
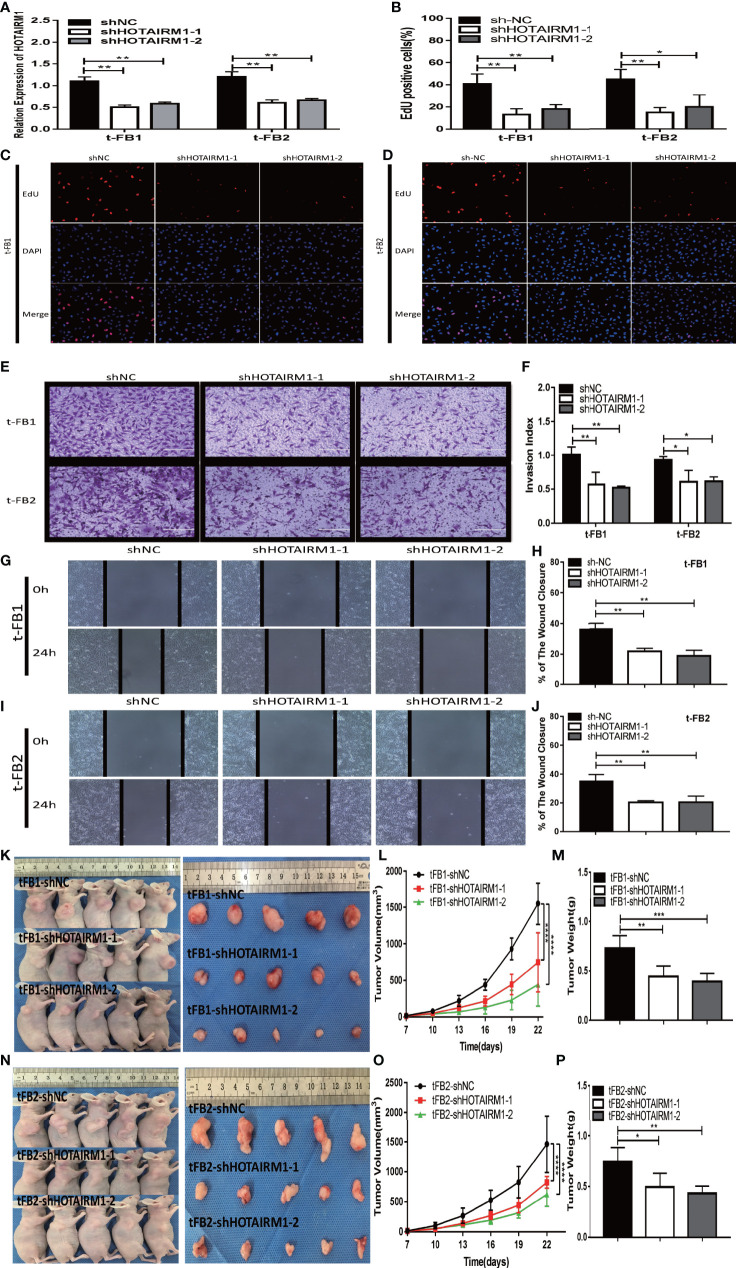
HOTAIRM1 downregulation resulted in inhibition on proliferation, invasion, and migration abilities of malignant transformed fibroblasts. **(A)** HOTAIRM1 expression was analyzed by qRT-PCR in t-FB1/2 transfected with shNC, shHOTAIRM1-1, or shHOTAIRM1-2. **p < 0.01, one-way ANOVA. **(B–D)** Proliferation ability was evaluated after HOTAIRM1 downregulation in t-FB1/2 using EdU assay. *p < 0.05, **p < 0.01, one-way ANOVA. **(E, F)** Invasion capacity was measured after HOTAIRM1 downregulation in t-FB1/2 by transwell assay. *p < 0.05, **p < 0.01, one-way ANOVA. **(G–J)** Migration capacity was detected after downregulation of HOTAIRM1 in t-FB1/2 by wound healing assay. **p < 0.01, one-way ANOVA. **(K, N)** Tumorigenicity assay performed in nude mice by subcutaneous inoculation of t-FB1/2 cells transfected with shNC, shHOTAIRM1-1, or shHOTAIRM1-2, respectively. **(L, M, O, P)** Tumor growth curve and tumor weight of shNC, shHOTAIRM1-1, and shHOTAIRM1-2 group. ****p < 0.0001, two-way ANOVA; *p < 0.05, **p < 0.01, ***p < 0.001, one-way ANOVA.

After transfected with an shRNA targeting HOTAIRM1 (shHOTAIRM1-1 and shHOTAIRM1-2) in t-FB1 and t-FB2 cells, the transfection efficiency was validated by qRT-PCR (**Figure 2A**). EdU assay indicated that HOTAIRM1 downregulation decreased the proliferation ability of t-FB1 and t-FB2 cells (**Figures 2B–D**). Transwell assay showed that knock-down HOTAIRM1 expression resulted in significant decline of invasion ability of t-FB1 and t-FB2 cells (**Figures 2E, F**). Downregulation of HOTAIRM1 also remarkably weakened migration ability of t-FB1 and t-FB2 cells (**Figures 2G–J**), as shown in wound healing assay. Tumorigenicity assay indicated that HOTAIRM1 downregulation of transformed fibroblasts led to obvious decrease in both tumor volume and weight of subcutaneous implanted HOTAIRM1 knocking-down t-FB1/2 cells decreased obviously, compared with the control group (**Figures 2K–P**). To further investigate the effect of HOTAIRM1 down-regulation in t-FB1 and t-FB2 cells on tumorigenicity ability of GSCs, *in vivo* combined inoculation of GSCs-SU3 and HOTAIRM1 downregulated t-FB1/2 cells was performed, the transplanted tumor volume and weight were significantly decreased, compared with the control group (**Supplementary Figures 2A–F**).

### HOTAIRM1 Upregulation Promoted Proliferation, Invasion, Migration, and Tumorigenesis of Malignant Transformed Fibroblasts

**Figure 3:**
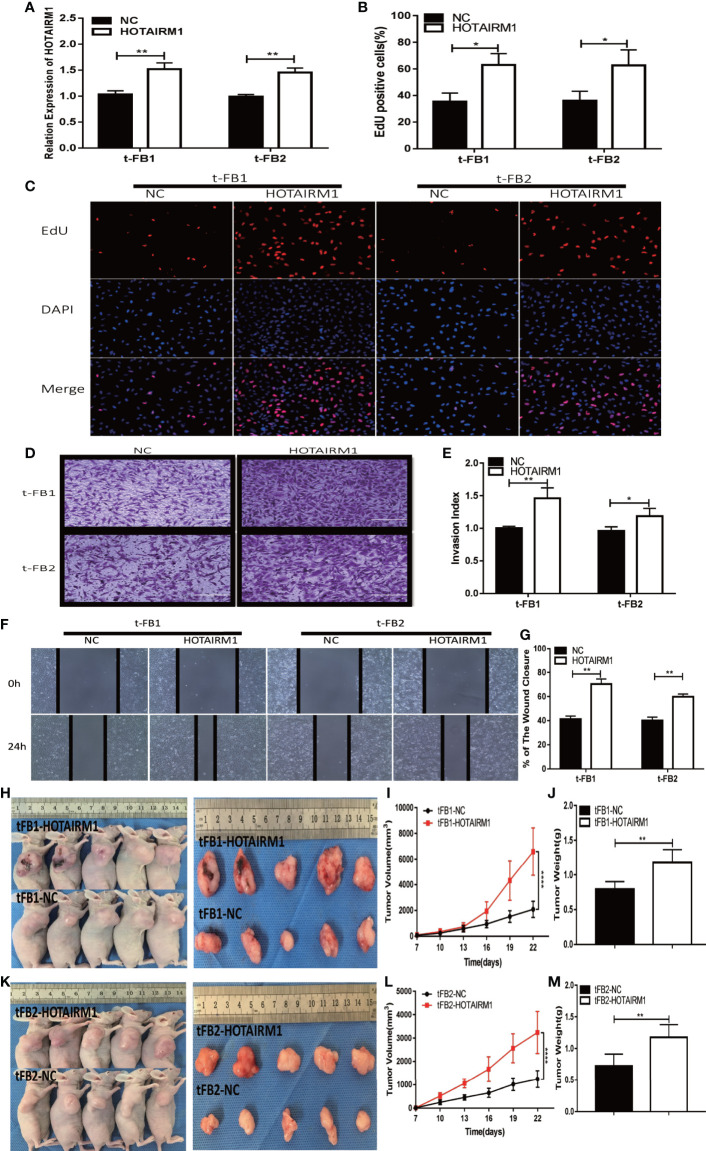
HOTAIRM1 upregulation promoted proliferation, invasion, and migration of malignant transformed fibroblasts. **(A)** HOTAIRM1 expression was analyzed with qRT-PCR in t-FB1/2 transfected with HOTAIRM1 or NC. **p < 0.01, Student’s t test. **(B, C)** Proliferation ability was evaluated after HOTAIRM1 upregulation in t-FB1/2 using EdU assay. *p < 0.05, Student’s t test. **(D, E)** Invasion capacity was assessed after HOTAIRM1 upregulation in t-FB1/2 by transwell assay. *p < 0.05, **p < 0.01, Student’s t test. **(F, G)** Migration ability was evaluated in t-FB1/2 with HOTAIRM1 overexpression by wound healing assay. **p < 0.01, Student’s t test. **(H, K)** Tumorigenicity was compared in murine subcutaneous tumor model between t-FB1/2 with NC or HOTAIRM1 transfection. **(I, J, L, M)** Tumor growth curve and tumor weight of NC and HOTAIRM1 transfection group. ****p < 0.0001, two-way ANOVA; **p < 0.01, one-way ANOVA.

To further validate the biological function of HOTAIRM1 in the malignant transformed fibroblasts, overexpression of HOTAIRM1in t-FB1 and t-FB2 cells was achieved *via* transfection of HOTAIRM1, and verified by qRT-PCR (**Figure 3A**). EdU assay showed HOTAIRM1 upregulation enhanced the proliferation capacity of t-FB1 and t-FB2 cells *in vitro* (**Figures 3B, C**). The results of Transwell assays indicated that overexpression of HOTAIRM1promoted the invasion of t-FB1 and t-FB2 cells (**Figures 3D, E**). Wound healing assay suggested that overexpression of HOTAIRM1 significantly increased the migration of t-FB1 and t-FB2 cells (**Figures 3F, G**). *In vivo* tumorigenicity assay also showed that overexpression of HOTAIRM1 in t-FB1/2 cells led to higher tumor volume and weight, compared with the control group (**Figures 3H–M**). To further investigate the effect of HOTAIRM1 overexpression of transformed fibroblasts on tumorigenicity of GSCs, *in vivo* combined inoculation of GSCs-SU3 and HOTAIRM1 upregulated t-FB1/2 cells was performed, tumor volume and weight were increased, compared with the control group (**Supplementary Figures 2G–L**).

### MiR-133b-3p Was a Direct Target of HOTAIRM1

**Figure 4:**
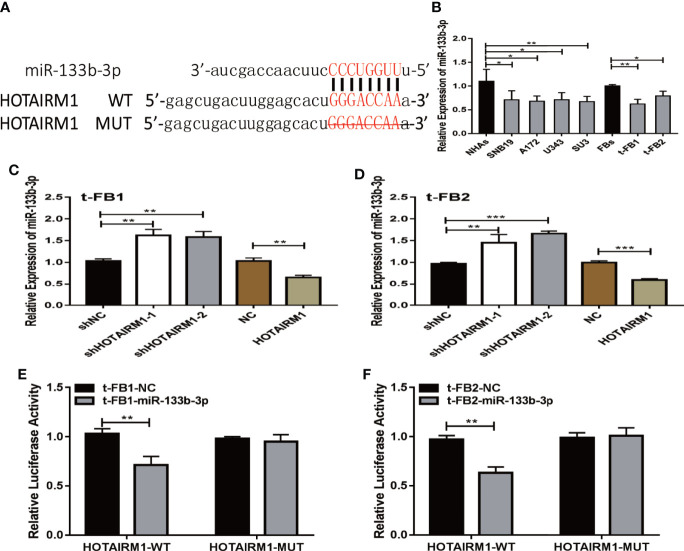
MiR-133b-3p was verified to be a direct target of HOTAIRM1. **(A)** the binding site between miR-133b-3p and HOTAIRM1. Wild type (WT) and mutant type (MT) vector of HOTAIRM1 were constructed for luciferase activity assay. **(B)** MiR-133b-3p expression level was evaluated by qRT-PCR in t-FB1/2 and glioma cell lines. *p < 0.05, **p < 0.01, one-way ANOVA. **(C, D)** qRT-PCR assay showed miR-133b-3p was negatively regulated by HOTAIRM1 in t-FB1/2. **p < 0.01, ***p < 0.001, one-way ANOVA; **p < 0.01, ***p < 0.001, Student’s t test. **(E, F)** Luciferase reporter assay suggested that miR-133b-3p weakened the luciferase activity of HOTAIRM1-WT. **p < 0.01, Student’s t test.

To explore the role of competing endogenous RNA (ceRNA) on regulating biological activities of malignant transformed fibroblasts, the online database starBase (http://starbase.sysu.edu.cn/) was applied to identify the possible miRNA targets of HOTAIRM1. Potential binding sites between HOTAIRM1 and miR-133b-3p (**Figure 4A**) were predicted with bioinformatic analysis *via* starBase. Further verification with qRT-PCR found dramatic downregulation of miR-133b-3p expression in t-FB1/2 and glioma cell lines, compared with normal fibroblasts and NHAs (**Figure 4B**). Specifically, miR-133b-3p was negatively regulated by HOTAIRM1 in t-FB1/2 cells (**Figures 4C, D**). In order to clarify the direct interaction between miR-133b-3p and HOTAIRM1, wild type (WT) and mutant type (MUT) vector of HOTAIRM1 were constructed for luciferase activity assay, which showed that miR-133b-3p significantly inhibited the luciferase activity of HOTAIRM1-WT (**Figures 4E, F**), indicating that miR-133b-3p was the direct target of HOTAIRM1.

### MiR-133b-3p Inhibited Proliferation, Invasion, and Migration of Malignant Transformed Fibroblasts by Targeting TGFβ

**Figure 5:**
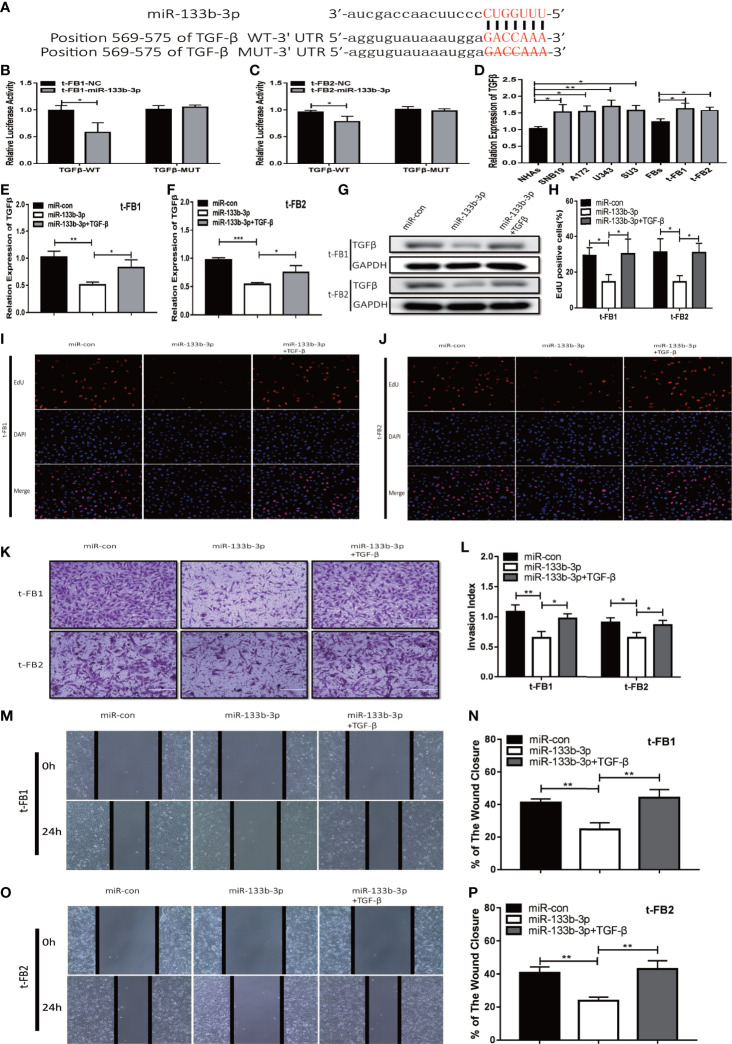
Upregulation of miR-133b-3p can inhibit proliferation, invasion, and migration of transformed fibroblasts by targeting TGFβ. **(A)** The vector of TGFβ-WT and TGFβ-MUT were constructed for luciferase activity assay. **(B, C)** Luciferase reporter assay testified that miR-133b-3p significantly weakened the luciferase activity of TGFβ-WT. *p < 0.05, Student’s t test. **(D)** TGFβ expression level in t-FB1/2 and glioma cell lines. *p < 0.05, **p < 0.01, one-way ANOVA. **(E–G)** qRT-PCR and western blot analysis of TGFβ expression in t-FB1/2 transfected with miR-133b-3p or miR-133b-3p together with TGFβ. *p < 0.05, **p < 0.01, ***p < 0.001, one-way ANOVA. **(H–J)** proliferation was analyzed in t-FB1/2 cells transfected with miR-133b-3p or TGFβ by EdU assay. *p < 0.05, one-way ANOVA. **(K, L)** Invasion was evaluated in t-FB1/2 cells transfected with miR-133b-3p or TGFβ *via* transwell assay. *p < 0.05, **p < 0.01, one-way ANOVA. **(M–P)** Migration was determined in t-FB1/2 transfected with miR-133b-3p or TGFβ by wound healing assays. **p < 0.01, one-way ANOVA.

TGFβ was one of the predicted downstream targets of miR-133b-3p according to bioinformatic analysis (**Figures 5A**). To further verify the direct bonding between miR-133b-3p and TGFβ, TGFβ-WT and TGFβ-MUT vectors constructed for further luciferase activity assay (**Figure 5A**), which revealed that miR-133b-3p significantly weakened luciferase activity of TGFβ-WT, compared with TGFβ-MUT (**Figures 5B, C**). TGFβ expression level was also detected with qRT-PCR in both glioma cell lines and t-FB1/2 cells, which showed TGFβ was overexpressed in both glioma cell lines and transformed fibroblasts (**Figure 5D**).

To further explore the regulatory relationship between miR-133b-3p and TGFβ, both of miR-133b-3p mimics and TGFβ plasmid were transfected into t-FB1/2 cells. QRT-PCR, western blot assay, and ELISA showed that miR-133b-3p can inhibit TGFβ expression, and this inhibition effect can be partially offset by TGFβ plasmid transfection (**Figures 5E–G**; **Supplementary Figures 3A, B**). In addition, EdU assay showed miR-133b-3p decreased t-FB1/2 cells proliferation, and can be reversed by TGFβ overexpression (**Figures 5H–J**). Meanwhile, the weakened invasion and migration of t-FB1/2 cells induced by miR-133b-3p upregulation were partially offset by TGFβ overexpression (**Figures 5K–P**).

### HOTAIRM1 Facilitated Progression of Malignant Transformed Fibroblasts by Regulating miR-133b-3p/TGFβ Axis

**Figure 6:**
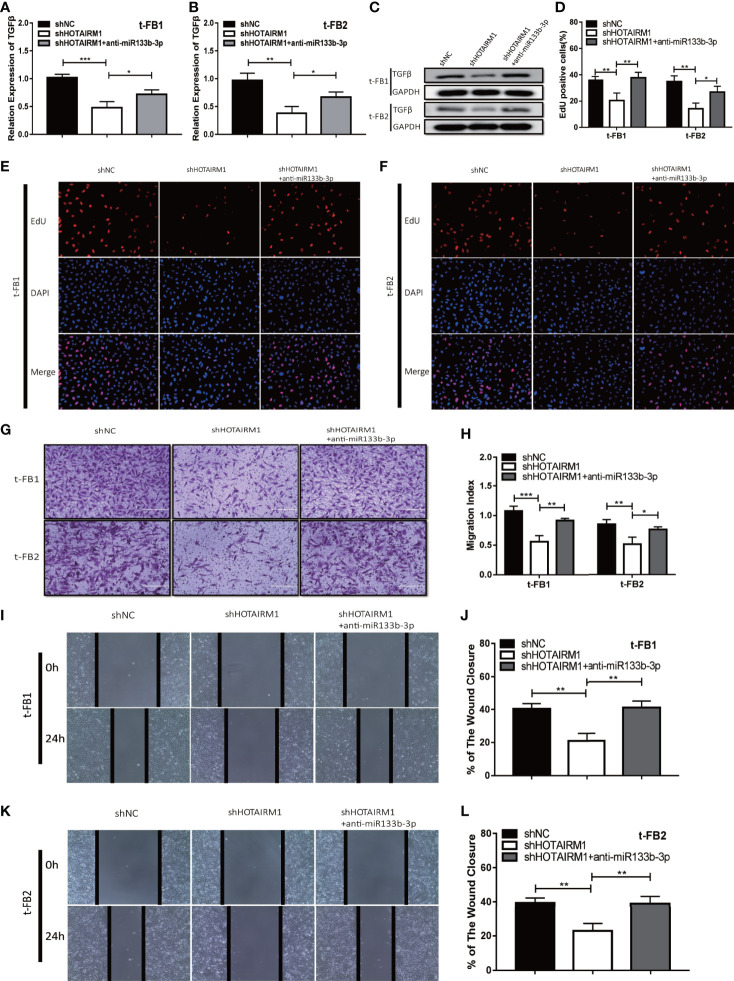
HOTAIRM1 facilitated malignant transformed fibroblasts progression *via* regulating miR-133b-3p/TGFβ axis. **(A–C)** qRT-PCR and western blot analysis of TGFβ expression in t-FB1/2 cells transfected with shHOTAIRM1 or shHOTAIRM1 together with miR-133b-3p inhibitors. *p < 0.05, **p < 0.01, ***p < 0.001, one-way ANOVA. **(D–F)** EdU assay was performed to evaluate the proliferation of t-FB1/2 cells transfected with shHOTAIRM1 or shHOTAIRM1 together with miR-133b-3p inhibitors. *p < 0.05, **p < 0.01, one-way ANOVA. **(G, H)** Invasion was evaluated in t-FB1/2 cells transfected with shHOTAIRM1 or shHOTAIRM1 together with miR-133b-3p inhibitors by transwell assay. *p < 0.05, **p < 0.01, ***p < 0.001, one-way ANOVA. **(I–L)** Migration was assessed in t-FB1/2 cells transfected with shHOTAIRM1 together with miR-133b-3p inhibitors by wound healing assay. **p < 0.01, one-way ANOVA.

To elucidate the mechanism which HOTAIRM1 promoted malignancy of transformed fibroblasts *via* regulating miR-133b-3p/TGFβ axis, t-FB1 and t-FB2 cells were transfected with shHOTAIRM1 or shHOTAIRM1 together with miR-133b-3p inhibitors. qRT-PCR, western blot assay, and ELISA suggested that shHOTAIRM1 can inhibit TGFβ expression, which was partly offset by miR-133b-3p inhibitors (**Figures 6A–C**; **Supplementary Figures 3C, D**). Besides, EdU assay indicated shHOTAIRM1 decreased the proliferation of t-FB1/2 cells, which could be reversed by miR-133b-3p inhibitors (**Figures 6D–F**). Consistent with results of EdU assay, the weakened invasion and migration of t-FB1/2 cells induced by shHOTAIRM1 were partly rescued by miR-133b-3p inhibitors as well (**Figures 6G–L**).

## Discussion

In this study, glioma associated fibroblasts were found undergoing malignant transformation in GSCs remodeled tumor microenvironment. The role of lncHOTAIRM1 was highly associated with the pro-tumor abilities of transformed fibroblasts, including promoting proliferation, invasion, migration, and tumorigenesis on t-FBs. Further mechanistical studies suggested that HOTAIRM1 competitively bound miR-133b-3p to regulate the expression of TGF-β, thus producing obvious pro-tumor effect.

Most studies attributed treatment resistance and high recurrence of glioma to GSCs (29–31), which based on their infinite self-renewal capacity, persistent proliferation, and strong remodeling of TME (32–34). Despite the key roles of GSCs in tumor initiation, glioma microenvironment, including the relevant various stromal cells, interacted actively with GSCs, and played vital roles on GSCs initiated tissue remodeling processes. Various glioma biological processes were largely affected by TME (35), and targeting on TME has been the potential treatment strategy in recent years (36). Among stromal cells in glioma microenvironment, fibroblasts were essential for regulating glioma development (37–39). Normal fibroblasts maintained homeostasis, which was characterized of inertness and low levels of synthetic properties if they were not under any local microenvironment stress (40). When the intrinsic crosstalk between normal fibroblasts (NFs) and stroma changed due to education by cancer stem cells, NFs may acquire modified phenotypes and can converted into CAFs (41). Recent studies revealed that CAFs were main cancer-promoting components in TME, which were involved in carcinogenesis, proliferation, invasion, and chemoresistance (42–45), and the existence of CAFs implied poor prognosis in ovarian and breast cancer (43, 44). Yes-associated protein 1 (YAP1) was responsible for converting NFs into CAFs, thus facilitating prostate cancer development (46). However, the role of fibroblasts in glioma microenvironment and how they interacted with GSCs kept largely unknown. *In vitro* studies showed CAFs were associated with glioma cell migration (38). Fibroblasts can differentiate from mesenchymal cells (47, 48), which can be recruited into glioma microenvironment. Our results verified malignant transformation of fibroblasts in glioma microenvironment induced by GSCs, and further disclosed that HOTAIRM1 played active roles on malignancy phenotype of t-FBs, which could be a potential target against gliomas.

Long non-coding RNAs were pivotal in molecular dysregulation of gliomas, including the aberrant expression profile of gliomas (49–51). Previous studies reported that Lnc HOTAIRM1 can act as promoter or suppressor in tumor development: HOTAIRM1 exerted its tumor inhibitory effect through competitive combination with endogenous RNA in gastric cancer (24), head and neck tumor (52), it also can suppress tumor cell proliferation and promote cell apoptosis in hepatocellular carcinoma *via* inhibiting Wnt pathway (53). Besides, HOTAIRM1 can promote glioma progression through certain ceRNA networks (54–57). The role of HOTAIRM1 in transformation and malignant phenotype of fibroblasts in glioma microenvironment was explored in the current studies, which also disclosed that miR-133b-3p was the target of HOTAIRM1 and can inhibit biological behaviors of t-FBs. MiR-133b played tumor suppressing roles in several malignancies through regulating in gastric cancer (58), targeting Sox9 in breast cancer (59), negatively regulating EMP2 in glioma (60), targeting methyltransferase DOT1L in colorectal cancer (61), and targeting EGFR in esophageal squamous cell carcinoma (62). Our results supported that TGFβ was the functional target of miR-133b-3p, miR-133b-3p can inhibit t-FBs *via* targeting TGFβ.

In summary, the current studies showed that HOTAIRM1 was upregulated in t-FB and gliomas, it can promote malignant biological phenotype of t-FBs by regulating TGFβ *via* miR-133b-3p. Therefore, the HOTAIRM1/miR-133b-3p/TGFβ signal axis may serve as the potential therapeutic target against t-FBs in glioma microenvironment. However, our results were mainly based on an orthotopic xenograft tumor model, which may not fully reflect the real TME of glioma patients, which needs to be verified through high throughput single cell sequencing of glioma surgical specimens to confirm the molecular mechanisms regulating CAFs in glioma microenvironment.

## Data Availability Statement

The raw data supporting the conclusions of this article will be made available by the authors, without undue reservation.

## Ethics Statement

The animal study was reviewed and approved by the ethics committee of Second Affiliated Hospital of Soochow University.

## Author Contributions

HW and JD conceived and designed the experiment. HW performed the experiments and wrote the manuscript. HL performed the statistical analyses and generated the figures. QJ and XD collected the public data. SL, SC, JS and LL collected the patient samples. ZQ and JD revised the manuscript. All authors contributed to the article and approved the submitted version.

## Funding

This study was supported by grants from National Natural Science Foundation of China (NO. 81472739), Research and Practice Innovation Program for Postgraduates in Jiangsu (No. KYCX19_1982), and Clinical Special Disease Diagnosis and Treatment Technology in Suzhou, China (No. LCZX201807).

## Conflict of Interest

The authors declare that the research was conducted in the absence of any commercial or financial relationships that could be construed as a potential conflict of interest.

## Supplementary Material

Supplementary Figure 1 Glioma stem cells can induce malignant transformation of fibroblasts. **(A)** Patient-derived GSCs-SU3 cells had typical sphere-like cell clusters. **(B)** SU3 cells had stable RFP expression after RFP transfection. **(C)** Orthotopic tumor formation after SU3-RFP cells inoculated in transgenic GFP BALB/c athymic nude mice. **(D–F)** Primary culture of the xenograft tumor cells and observation under the fluorescence microscope. **(G, H)** GFP+ cells with high proliferation ability were mono-cloned in 96-well plates. **(I, J)** Subculture of the mono-cloned GFP+ cells. **(K, L)** the unlimited proliferation ability of GFP+ cells was measured by CCK8 assay. **(M)** GFP+ cells with high proliferation ability were positive for fibroblasts makers (α-SMA, vimentin, and S100A4).

Supplementary Figure 2 Tumorigenicity assay by subcutaneous inoculation of GSCs-SU3 cells, and t-FB1/2 cells with HOTAIRM1 down-regulation or overexpression. **(A, D)** Tumorigenicity was compared in GSCs subcutaneous tumor model after simultaneous inoculation of t-FB1/2 cells with shNC, shHOTAIRM1-1, or shHOTAIRM1-2 transfection. **(B, C, E, F)** Tumor growth curve and tumor weight of shNC, shHOTAIRM1-1, and shHOTAIRM1-2 group. ****p < 0.0001, two-way ANOVA; **p < 0.01, one-way ANOVA. **(G, J)** Tumorigenicity was compared in GSCs subcutaneous tumor model after simultaneous inoculation of t-FB1/2 cells with NC or HOTAIRM1 transfection. **(H, I, K, L)** Tumor growth curve and tumor weight of NC and HOTAIRM1 transfection group. ****p < 0.0001, two-way ANOVA; ***p < 0.001, Student’s t test.

Supplementary Figure 3 ELISA to detect TGFβ secretion. **(A, B)** Secretion of TGFβ by t-FB1/2 cells which were transfected with miR-133b-3p or miR-133b-3p together with TGFβ. **(C, D)** Secretion of TGFβ by t-FB1/2 cells which were transfected with shHOTAIRM1 or shHOTAIRM1 together with miR-133b-3p inhibitors. *p < 0.05, **p < 0.01, one-way ANOVA.
