# Supplementary figures and images for: HOTAIRM1 Promotes Malignant Progression of Transformed Fibroblasts in Glioma Stem-Like Cells Remodeled Microenvironment via Regulating miR-133b-3p/TGFβ Axis
Source: Front Oncol. 2021 Mar 19;11:603128. doi: 10.3389/fonc.2021.603128 (PMC8017308; doi:10.3389/fonc.2021.603128)

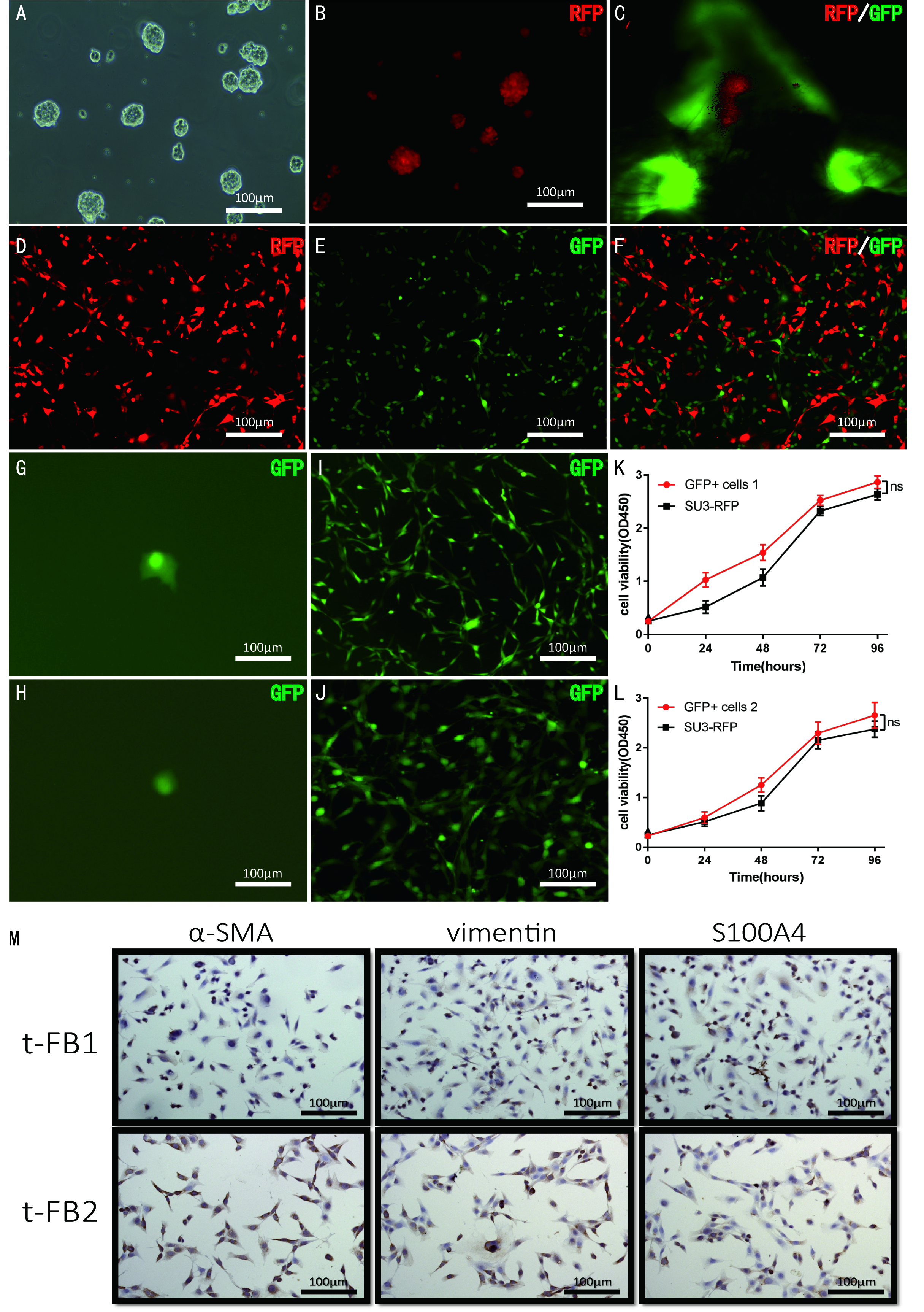

Supplement: Supplementary Figure 1 — Glioma stem cells can induce malignant transformation of fibroblasts. (A) Patient-derived GSCs-SU3 cells had typical sphere-like cell clusters. (B) SU3 cells had stable RFP expression after RFP transfection. (C) Orthotopic tumor formation after SU3-RFP cells inoculated in transgenic GFP BALB/c athymic nude mice. (D–F) Primary culture of the xenograft tumor cells and observation under the fluorescence microscope. (G, H) GFP+ cells with high proliferation ability were mono-cloned in 96-well plates. (I, J) Subculture of the mono-cloned GFP+ cells. (K, L) the unlimited proliferation ability of GFP+ cells was measured by CCK8 assay. (M) GFP+ cells with high proliferation ability were positive for fibroblasts makers (α-SMA, vimentin, and S100A4). [file Image_1.tif]

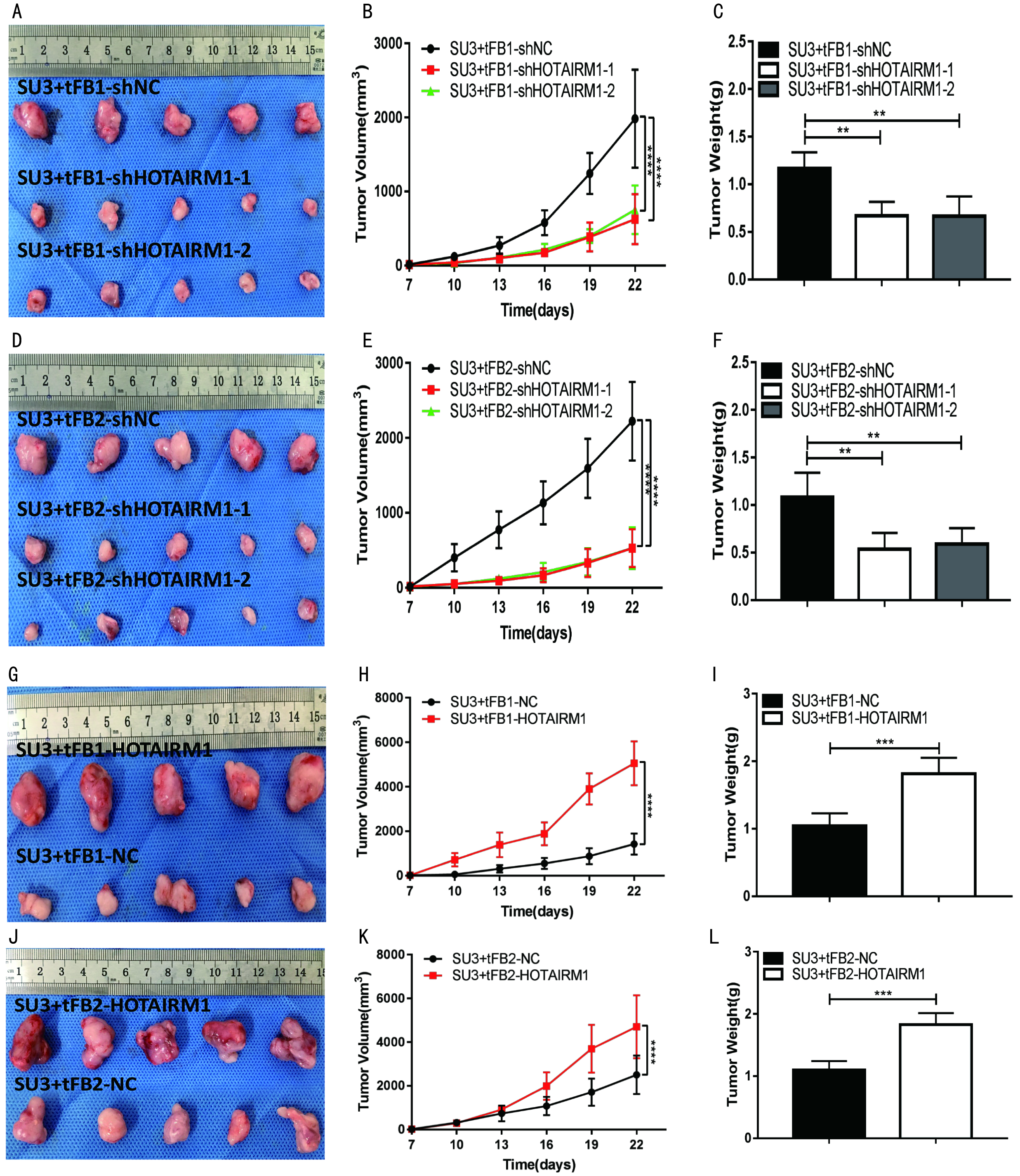

Supplement: Supplementary Figure 2 — Tumorigenicity assay by subcutaneous inoculation of GSCs-SU3 cells, and t-FB1/2 cells with HOTAIRM1 down-regulation or overexpression. (A, D) Tumorigenicity was compared in GSCs subcutaneous tumor model after simultaneous inoculation of t-FB1/2 cells with shNC, shHOTAIRM1-1, or shHOTAIRM1-2 transfection. (B, C, E, F) Tumor growth curve and tumor weight of shNC, shHOTAIRM1-1, and shHOTAIRM1-2 group. ****p < 0.0001, two-way ANOVA; **p < 0.01, one-way ANOVA. (G, J) Tumorigenicity was compared in GSCs subcutaneous tumor model after simultaneous inoculation of t-FB1/2 cells with NC or HOTAIRM1 transfection. (H, I, K, L) Tumor growth curve and tumor weight of NC and HOTAIRM1 transfection group. ****p < 0.0001, two-way ANOVA; ***p < 0.001, Student’s t test. [file Image_2.tif]

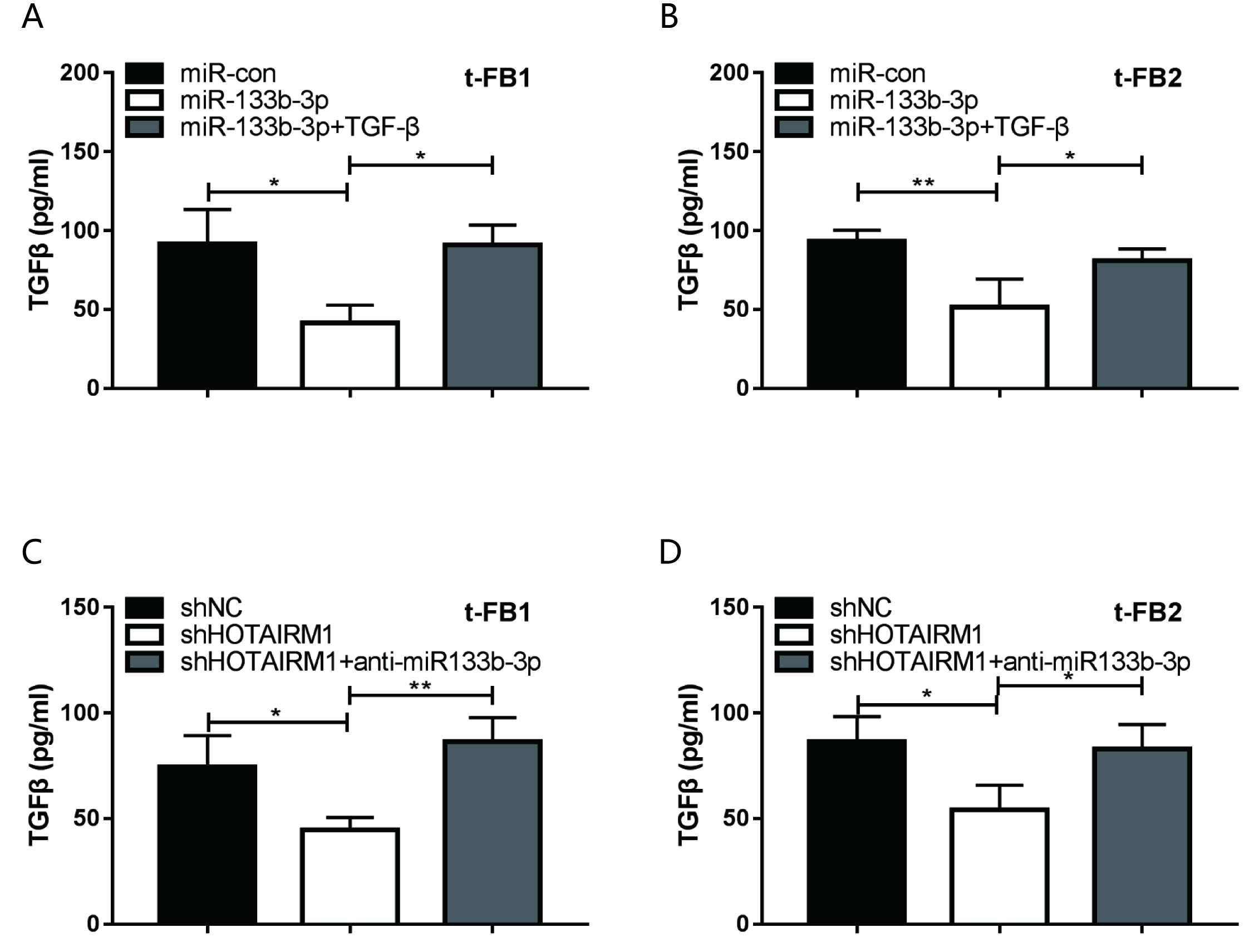

Supplement: Supplementary Figure 3 — ELISA to detect TGFβ secretion. (A, B) Secretion of TGFβ by t-FB1/2 cells which were transfected with miR-133b-3p or miR-133b-3p together with TGFβ. (C, D) Secretion of TGFβ by t-FB1/2 cells which were transfected with shHOTAIRM1 or shHOTAIRM1 together with miR-133b-3p inhibitors. *p < 0.05, **p < 0.01, one-way ANOVA. [file Image_3.tif]
